# Influence of body mobility on attention networks in school-aged prematurely born children: A controlled trial

**DOI:** 10.3389/fped.2022.928541

**Published:** 2022-09-08

**Authors:** Joëlle Rosenbaum, Hadrien Ceyte, Isabelle Hamon, Hélène Deforge, Alexandre M. J. Hascoët, Sébastien Caudron, Jean-Michel Hascoët

**Affiliations:** ^1^Développement, Adaptation et Handicap Laboratory (DevAH), Université de Lorraine, Nancy, France; ^2^Aix-Marseille University, CNRS, ISM, Marseille, France; ^3^CHRU, Maternité Régionale, Nancy, France; ^4^CNRS, LPNC, Université Grenoble Alpes, Université Savoie Mont Blanc, Grenoble, France

**Keywords:** prematurity, posture, body mobility, alertness, orienting, executive control

## Abstract

School-aged prematurely born children (PC) have a higher risk of academic difficulties, which may be partly explained by attention difficulties. It has been suggested that children’s attentional performance might be influenced by their body posture and spontaneous body motion. The aim of this study (ClinicalTrials.gov – NCT 03125447) was to test the influence of three body mobility conditions on the three functions of attention (alertness, orienting, and executive control) among school-aged PC vs. term-born children (TC). Notably, 21 PC and 21 TC performed the Attention Network Test for Children in three body mobility conditions, namely, sitting and standing imposed fixed postures and a free-to-move condition. The children’s median reaction times were compared between trials (1) with and without alerting cues, (2) with valid and invalid orienting cues, and (3) with and without distracting information, to calculate the performance of alertness, orienting, and executive control, respectively. Results showed that with distracting information, PC exhibited significantly slower responses in the standing-still posture than in the sitting-still posture (1,077 ± 240 vs. 1,175 ± 273 ms, *p* < 0.05), but not TC. No difference was observed with the free-to-move condition. PC and TC did not significantly differ in alertness or orienting, regardless of body mobility condition. These data suggest that PC must use executive resources to stand still and maintain position, which impairs their performance during executive tasks. We speculate that these results may be related to less developed postural control and motor inhibition in PC.

## Introduction

Prematurely born children (PC) carry a risk of adverse long-term outcomes (1–3) including a high risk of cognitive impairment at school age (1, 4). Academic difficulties are more frequent among PC than in term-born children (TC), as revealed by lower reading, spelling, and mathematics performance, and greater needs for special education (5–7). These academic difficulties are sustained throughout schooling, suggesting that it is difficult to catch up from early learning delays (5, 8).

Learning difficulties in PC are partly explained by poorer executive functions and attention abilities (9–13). In fact, PC exhibit lower performance in attention skills during childhood (14–21). Three attentional functions have been described: *alertness*, *orienting*, and *executive control* (22). *Alertness* refers to the achievement and maintenance of an appropriate level of vigilance. It is divided into the general control of arousal (*tonic alertness*) during a task and the increase in response readiness following an acute external cueing *(phasic alertness)* (23). Although this function strongly developed at preschool age (24), at 6 years of age, tonic and phasic alertness have not reached the adult level yet (25). *Orienting* refers to the ability to select and prioritize information from the environment (23). Some studies indicate that automatic attention shifting is mature at 6 years, (25) while others suggest later development (26, 27). Finally, *executive control*, also termed *inhibition*, corresponds to the ability to detect errors, resolve conflicts, and resist distraction during goal-oriented behaviors. This develops strongly in preschool-aged children and continues to advance throughout childhood (19, 28–30).

The efficiency of each attentional function may be separately assessed using the Attention Network Test (ANT), (31) which has been adapted for children (ANT-c) (25). Studies have used this test to describe attentional functions during childhood among TC (32) and PC (14, 15, 17). While overall response time and accuracy may be reduced in PC compared with TC (14, 15) due to more lapses of attention (14), the ANT-c highlights that prematurity may have different effects on each attentional function (14, 15, 17, 19). In fact, while PC and TC of 5–7 years of age exhibit no significant differences in phasic alertness (14–17) or exogenous orienting (14, 15), PC show poorer executive performance (15, 17). This suggests that PC have greater difficulties in maintaining attention for a long time and resisting distraction in class.

School-aged children have a substantial urge for mobility (33) and are sometimes described as “hyperactive” even in the absence of significant attention disorders, which is thus different from attention-deficit/hyperactivity disorder syndrome. Additionally, this excessive motor activity may be transient (34) during childhood, raising questions about its role in young children. Notably, children’s motor activity does not always disturb attention functions, as revealed by the improvement of tonic alertness and executive control following physical activities (35, 36). The relationship between motor and cognitive activities strongly depends on the body posture, the motor activity, and the cognitive function studied. For example, adults have performed better on arithmetic tasks in a sitting posture than in a standing posture, whereas they performed better on memory tasks while walking (37). Regarding the function of attention, alertness (38, 39), and to a lesser extent executive control (40–43), but not orienting (38), may be improved in a standing compared with a sitting posture. We also previously demonstrated that in TC of 6–7 years of age, standing may improve executive control compared with a sitting posture (32). Spontaneous body motions and adopting a standing/upright posture may enable children to increase cortical arousal (40, 44–46). This beneficial influence of changing posture (38, 40) or of physical activity (35, 36) on attention suggests that the classic “sitting still posture” at school may not be optimal for children’s attention. Free mobility might be more effective than an imposed mobility or posture, as suggested by the increased memory performance when children walked at their preferred speed, but not at an imposed speed (47).

In this study, the primary aim was to determine the influence of posture and free mobility on the three attentional functions in PC vs. TC. We hypothesized that adopting free mobility, as compared with imposed postures, might help PC improve their attention performance. Additionally, we hypothesized that a standing posture would improve attention performance, as compared with a sitting posture.

## Materials and methods

This prospective controlled study was performed at the Pediatric Outpatient Unit of the level III Maternity Hospital of Nancy. It was conducted in accordance with the Declaration of Helsinki and approved by the Comité de Protection des Personnes Sud-Est III Ethics Committee (2017–010 B). The method of this study has already been documented, (48) and TC behavior was previously analyzed (32).

In brief, inclusion criteria were PC with gestational age (GA) of <34 weeks, aged between 6 and 7 years. These children were included in a routine regional follow-up program. They were thoroughly followed yearly for neurodevelopment and motor ability. Only infants without disability or previously diagnosed neurodevelopment delay were involved in this study. They were attending normal schools at their appropriate grade level.

Children with any neurological, cognitive, developmental, or motor disorders preventing the realization of the tests were excluded. Exclusion criteria included any motor impairment, visual refractory impairment, strabismus, visuospatial difficulties not corrected by orthoptic therapy, daltonism, not corrected hearing loss, suspected or diagnosed attention deficit hyperactivity disorder, or autism spectrum disorder.

To recruit TC of equal age, parents were informed about the study through leaflets. Sociodemographic, perinatal, morbidity, anthropometric data, and visual acuity were recorded by a certified trained pediatrician during a clinical examination. Since PC are usually considered to have a learning ability delay of approximately 1 year, we previously determined (48) that we would need 24 children in each group to demonstrate some catch-up in PC attention performance related to body mobility condition, with sitting posture as the reference, with an alpha risk of 0.00625 (Bonferroni correction for the number of tests) and a power of 0.80 (Power and Precision V4, Biostat Inc., Englewood, NJ, United States 2001). Additionally, as a secondary evaluation of the effect of postnatal age, we stratified the children into two groups according to the median age of our population.

### Materials and design

#### The attention network test for children

Children were instructed to click as quickly as possible on the right or left button of a mouse, depending on whether a target fish was facing left or right (25). This target could appear above or below a central cross, alone or surrounded by flanking fishes pointing toward the same (congruent) or the opposite (incongruent) direction. Furthermore, it could appear suddenly (no cue) or be preceded by one of three equiprobable warning cues (an asterisk appearing 150 ms before the fish): a spatial cue or a center cue orienting attention toward the location of the upcoming target or the central cross, respectively, or a double cue giving no directional information.

#### Body mobility condition

Children performed the ANT-c in three pseudo-randomized body mobility conditions: (i) sitting-still posture, (ii) standing-still posture, and (iii) free-to-move condition. The ANT-c was generated using the E-Prime software (version 3.0 professional; Psychological Software Tools^®^, Sharpsburg PA, United States) and was projected with a head-mounted display (iWear Video Headphones, The Way In^®^, Vuzix Corporation, New York, United States) to keep the child’s eyes and the target constant, in any body mobility condition. Children’s movements were video-recorded, reported on a standardized evaluation grid, and independently analyzed by JR and HC. The general amount of movements (Mov) was calculated for each body mobility condition (32).

For each body mobility condition, a block of 48 trials was performed, with 3 min of rest between blocks. The experiment lasted approximately 45 min. Children’s success and reaction times were recorded for each trial.

### Data reduction and statistical analyses

Normally distributed data are presented as mean values and standard deviation (M ± SD); others as the median and interquartile range (Med [IQR]). A chi-square test was used to compare categorical variables between PC and TC.

For each child, we calculated the general success rate and the general median reaction time for correct responses (General RT) to determine the overall performance. The Mann-Whitney *U* tests were then performed on the general success rate to compare PC and TC in each body mobility condition, and a Friedman test was used to assess the effect of body mobility condition. In addition, mixed ANOVAs were performed on General RT, with the body mobility condition as a within-subject factor and prematurity (PC and TC) as a between-subject factor.

For each child, we also calculated the median reaction time (MedRT) for correct responses for the trials with the same condition of cue (no cue, double cue, center cue, or spatial cue) or the same condition of targets (congruent or incongruent). Then, the children’s performance in *challenging* trials (i.e., no cue, center cue, and incongruent trials) was compared with *control* trials (i.e., double cue, spatial cue, and congruent trials) for alertness, orienting, and executive control, respectively. Three mixed ANOVA were performed on MedRT (one for each attentional function), with prematurity as a between-subject factor and body mobility condition and challenging vs. control trials as within-subject factors. For these analyses, the children’s postnatal age was added as a covariate (49).

Additionally, to better evaluate the effect of development, we divided the children into two age groups separated by the medians of PC and TC, i.e., postnatal age of < 80 months vs. ≥ 80 months. They were evaluated by mixed ANOVA on MedRT, with the body mobility condition and the executive control targets as within-subject factors and the age group as a between-subject factor.

For all analyses, *post hoc* tests were conducted using Tukey’s honestly significant difference method.

## Results

Results among TC have already been described (32). The results among PC are presented below.

A total of 24 PC and 25 TC were eligible for this study. Three PC and three TC were unable to complete the experiment, and one TC made 51% of errors. Overall, 21 PC and 21 TC were included in our analyses. One PC responded correctly only once on all incongruent trials, despite further explanations and training. This child was included for analyses of alertness and orienting because her success rate was consistent with the group in all trials when incongruent trials were excluded. If some trials were missing, due to technical issues (*N* = 2) or children’s non-compliance (*N* = 2, range of missing trials rate: 5–26%), general success rate, GeneralRT, and MedRT were calculated from the remaining trials.

Table 1 summarizes the children’s characteristics. No significant differences were observed between the two groups, except for parents’ employment, as the mothers of PC were more likely to be unemployed or employees/workers than the mothers of TC (*p* = 0.002).

**TABLE 1:** Characteristics of preterm-born children and term-born children (included vs. non-included).

	Included		Non-included	
	Preterm	Term	Preterm	Term
*N*	21	21	3	4
Girls/Boys (*N*)	7/14	11/10	0/3	1/3
Multiple births	4**	/	1**	/
Gestational age, weeks (Mean ± SD)	29.6 (2.6)	39.2 (1.3)	27.6 (1.4)	40.3 (1.5)
Birth weight, g (Mean ± SD)	1318 (496)	3301 (383)	859 (157)	3657 (171)
Birth weight, *z*-score (Mean ± SD)	−0.23 (0.96)	−0.18 (0.62)	−0.89 (1.75)	0.08 (0.40)
IVH (*N*)	4*	/	1	/
Periventricular leukomalacia (*N*)	1	/	/	/
Age, months (Mean ± SD)	80.5 (5.3)	80.3 (6.8)	76.3 (5.1)	77.8 (5.9)
Corrected age, months (Mean ± SD)	78.1 (5.6)	80.3 (6.8)	73.4 (5.0)	77.8 (5.9)
BMI at assessment, kg/m^2^ (Mean ± SD)	15.1 (1.6)	14.9 (1.4)	15.5 (0.5)	15.0 (2.0)
Left-handed/Right-handed (*N*)	3/18	2/19	0/3	0/4
Bilateral hearing loss (*N*)	1	0	0	0
Parents in relationship (*N*)	17	20	2	4
Mother (*N*); Father (*N*) employment				
*Unemployed*	3; 0	0; 0	0; 0	0; 0
*Employees, workers*	5; 5	0; 1	1; 0	0; 1
*Farmers, artisans, shopkeepers*	0; 2	1; 1	0; 0	0; 0
*Intermediate professions*	8; 7	12; 7	2; 2	2; 1
*Executives, Intellectual*	5; 5	8; 12	0; 0	2; 2
**Child school grade (*N*)**				
*Preschoolers*	6	4	0	1
*First grade*	10	10	2	3
*Second grade*	5	7	1	0
Grade repetition (*N*)	1***	0	0	0
Special need support (*N*)	1	0	0	0

*IVH including grade I (N = 2), grade II (N = 1), and grade IV (N = 1).

**Including three births of twins (for one dyad, one twin was included and the other excluded) and one birth of triplets.

***Visuospatial dyspraxia. IVH, intraventricular hemorrhage.

### Overall performance (General RT and accuracy)

PC and TC did not differ in the general success rate, either for the sitting-still posture (95.8 [8.3] vs. 95.8 [8.3]%, *p* = 0.68), the standing-still posture (92.7 [11.0] vs. 95.8 [4.2]%, *p* = 0.12), or the free-to-move condition (91.7 [7.3] vs. 93.8 [4.2]%, *p* = 0.22). We also did not observe differences between the body mobility conditions. Table 2 presents the general RT for TC and PC. We found no main effect of prematurity (*p* = 0.17), body mobility (*p* = 0.89), or prematurity*body mobility interaction (*p* = 0.80).

**TABLE 2:** Mean (SD) of MedRT (in ms) for PC and TC for the different cues and targets, in the three body mobility conditions.

	Sitting		Standing		Free		All conditions	
**General RT**								

TC	930 (207)		919 (190)		937 (201)		929 (197)	
PC	997 (204)		1012 (203)		1009 (178)		1006 (192)	
TC and PC	964 (204)		966 (200)		973 (191)			

**Alertness**	**No**	**Double**	**No**	**Double**	**No**	**Double**	**No**	**Double**

TC	993 (205)	911 (260)	1002 (175)	905 (267)	996 (209)	934 (228)	997 (194)	917 (248)
PC	1031 (190)	976 (225)	1134 (211)	1038 (284)	1047 (182)	1020 (178)	1071 (197)	1011 (231)
TC and PC	1012 (196)	943 (242)	1068 (203)	972 (281)	1021 (196)	977 (206)	1034(198)*	964(243)*

**Orienting**	**Center**	**Spatial**	**Center**	**Spatial**	**Center**	**Spatial**	**Center**	**Spatial**

TC	902 (191)	916 (206)	902 (210)	921 (199)	944 (205)	888 (218)	916 (200)	908 (205)
PC	1004 (212)	1019 (223)	1008 (233)	956 (201)	998 (192)	1021 (230)	1003 (209)	999 (217)
TC and PC	953 (206)	967 (218)	955 (225)	938 (198)	971 (198)	955 (231)	960 (254)	954 (215)

**Executive control**	**Incongruent**	**Congruent**	**Incongruent**	**Congruent**	**Incongruent**	**Congruent**	**Incongruent**	**Congruent**

TC	1045 (260)	894 (199)^‡^	1001 (214)	906 (177)^‡^	1010 (238)	930(211)^‡^	1019 (235)	910 (193)
PC	1077 (240)^†^	972 (223)^‡^	1175 (293)^†^	1012 (249)^‡^	1097 (224)	967(191)^‡^	1116 (254)	983 (219)
TC and PC	1061 (248)	932 (212)^‡^	1086 (268)	958 (219)^‡^	1053 (233)	948(200)^‡^	1066 (248)	946(209)^‡^

^†^p < 0.05; *p < 0.01; ^‡^p < 0.05 incongruent vs. congruent. RT, reaction time; TC, term-born children (N = 21); PC, prematurely born children (N = 21); TC and PC, mean of MedRT for all children (N = 42).

### Separate attentional functions

Table 2 presents MedRT for PC and TC for the different conditions. The attention effects are presented in the supplemental material (Supplementary Figure 1) and can be calculated from the data in Table 2.

#### Executive control

**FIGURE 1:**
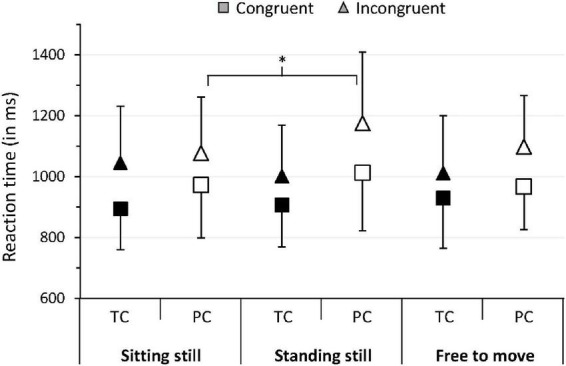
Mean of median RT for PC and TC in trials with congruent and incongruent targets in the three body mobility conditions. Error bars represent the mean absolute difference. *p < 0.05. TC, term-born children; PC, prematurely born children.

Our analyses revealed a significant body mobility*prematurity*target interaction effect for MedRT (*p* = 0.04, η^2^ = 0.08, 95%CI [0–0.21]). *Post hoc* analyses showed that PC and TC responded significantly faster in congruent trials compared with incongruent trials under the three body mobility conditions (*p* < 0.05, Table 2). However, during incongruent trials, PC responded significantly slower in the standing-still condition than in the sitting-still condition (*p* < 0.05) and tended to respond slower in the standing-still condition than in the free-to-move condition (*p* = 0.06), with no difference between the sitting-still vs. free-to-move condition (*p* > 0.05). Among TC, we found no significant difference in MedRT between the three body mobility conditions during incongruent trials. Moreover, among TC, we previously showed that the difference between incongruent and congruent trials was more important in the sitting-still posture than in the standing-still posture (32), suggesting that the sitting-still and standing-still postures have opposite effects between PC and TC. These results are summarized in Figure 1.

#### Alertness

All children (PC and TC) responded significantly faster with a double cue than without a cue (964 ± 243 vs. 1,034 ± 198 ms, *p* < 0.001, 95%CI [0.19–0.60]). This effect did not interact neither with body mobility (*p* < 0.11) with prematurity (*p* = 0.51) nor with body mobility by prematurity group (*p* = 0.51).

#### Orienting

The MedRT of all children (PC and TC) did not differ significantly between the two types of the orienting cue (954 ± 215 ms for the center cue vs. 960 ± 208 ms for the spatial cue, *p* = 0.61). This result neither interacts with body mobility (*p* = 0.89) nor with prematurity (*p* = 0.61). There was a significant body mobility*prematurity*cue interaction for MedRT (*p* = 0.03), but no difference was found on *post hoc* analyses.

### Age

For all attentional functions, age did not significantly interact with a cue or with the interaction of interest (all *p* > 0.05). When the children were divided according to median postnatal age of < or ≥ 80 months, we observed a significant main effect of age group on MedRT (*p* = 0.03, η^2^ = 0.11, 95%CI [0–0.31]), with younger children (74.7 ± 2.5 months) responding significantly slower than older children (85.0 ± 3.5 months, 1,081 ± 241 vs. 947 ± 217ms, respectively). However, age*target interaction (*p* = 0.89), or age*target*body mobility interaction (*p* = 0.37) did not reach significance.

### Movements of children during the ANT-c

The rates of head and limb movement for PC and TC in the three body mobility conditions are available in the supplemental material (Supplementary Figure 2). For all body mobility conditions and body parts, in both TC and PC, we found an over 80% rate of < 5 s of movement per minute. There was a significant effect of body mobility condition on Mov (W = 0.36; *p* < 0.001.). Mov was greater in the free-to-move condition than in the sitting-still condition (0.26 [0.84] vs. 0.09 [0.17], *r* = 0.85, *p* < 0.001) and the standing-still condition (0.07 [0.18], *r* = 0.81, *p* < 0.001), with no difference between the sitting-still and the standing-still conditions (*p* = 0.40). We found no difference of Mov between PC and TC in the sitting-still (0.12 [0.26] vs. 0.07 [0.15], *p* = 0.11), standing-still (0.08 [0.21] vs. 0.07 [0.13], *p* = 0.65), or free-to-move condition (0.53 [0.83] vs. 0.12 [0.50], *p* = 0.06).

## Discussion

### Overall performance

This study aimed to determine the influence of three body mobility conditions on the attention functions of prematurely and term-born children aged 6–7 years. It showed that, while no difference between PC and TC in terms of overall performance was found in any mobility condition, differences appeared when attentional functions were studied separately. Contrary to our *a priori* hypothesis, free mobility did not improve the attentional functions of PC or TC, as compared with fixed imposed postures. Nevertheless, compared with the sitting-still posture, adopting a standing-still posture resulted in decreased executive control for PC but not for TC.

### Separate attentional functions

#### Executive control

Prematurely born children exhibited reduced executive performance when standing still. On the opposite, we previously showed an improvement of executive control in TC when standing still, which we explained by an increase in arousal and optimal mental state in this posture (32). Olivier et al. suggested that interdependency between postural and executive activities may vary during childhood and depend on the level of difficulty of both cognitive and postural tasks (50). In line with this idea, we speculate that the standing-still posture was a more challenging postural task for PC. In fact, PC presents an increased risk of developmental coordination disorder (DCD). Studying 22,989 children from the Danish National Birth Cohort Questionnaire, Zhu et al. observed a significant inverse association between gestational age at birth and the risk of DCD at 7 years of age (51). DCD can be defined as minor gross and/or fine motor impairments, which cannot be explained by cerebral palsy or intellectual impairments but can influence the daily lives of children. In a meta-analysis of 36 studies, Van Horn et al. did not find any specific risk factor of DCD but preterm birth and male sex (52). In our study, despite no major motor coordination impairment was observed, one cannot exclude that some minor coordination disorder had contributed in part to the difficulties observed in PC when standing. In a study comparing 105 prematurely born infants to controls matched for age and sex with the Movement Assessment Battery for Children (M-ABC), De Rose et al. showed a higher risk of perceptual-motor difficulties at 4 years and concluded that a delayed maturation was possibly explaining the observed results (53). In line with our assumption that standing still is difficult for PC, other authors reported lower performance for these children during the balance tasks of the M-ABC, with greater difficulties standing on one leg or walking heel-to-toe (54–58). Also, some studies using a force plate (59–61), but not all (58, 62), revealed less efficient postural control with more body sway in PC. Lorefice et al., especially, reported more postural sway in 4 years PC than in TC when performing a picture naming task (59), supporting the functional link between postural and cognitive activities postulated by Olivier et al. (50). Thus, we proposed that the standing-still posture was difficult to maintain for PC, requiring executive resources, which decreased their performance in the trials demanding executive control. One may speculate that it was the cognitive and motor constraints associated with the instruction to stay still that made standing up very difficult for PC, as they may have difficulties suppressing automatic or inappropriate motor responses due to motor inhibition difficulties (19, 63).

This last assumption implies that the instruction “stay still without moving” may be difficult for PC and alter their performances. Nevertheless, the lack of executive control difference between the free-to-move condition and the sitting posture with the instruction to stay still might support that the posture adopted (standing vs. sitting) may more influence executive performance than the instruction to stay still. Supporting this view was that in the free-to-move condition, children often adopted a sitting posture [21 TC and 18 PC seated at least one time, among them 11 TC and 4 PC exclusively sat (Supplementary Figure 2 in Supplementary material)]. However, this ecological free-to-move condition where children (1) did not have the instruction to stay still and (2) could adopt a free motor behavior did not let us precisely disentangle the influence of the instruction from the influence of the posture and body mobility adopted on the executive performance of children. Further analysis may be interesting in order to respond to this answer.

#### Alertness

In all body mobility conditions, PC and TC were both faster after a warning signal, with PC achieving a level of readiness comparable with TC. This is consistent with the literature evaluating the phasic alertness of PC (14, 15, 17). Notably, the short duration of each block of trials did not allow the evaluation of tonic alertness in our study. Body mobility condition did not affect alertness in PC or TC, in contrast to studies of adults showing improved alertness when standing (38) or during physical activity (64). Children may not exhibit improved alertness when standing because it is more challenging for them to stand still than adults since their postural control is not fully developed (65). Regarding the free-to-move condition, one may speculate that the amount of children’s motor activities was not high enough to increase arousal to a sufficient level for improving alertness (45).

#### Orienting

Prematurity was not found to influence orienting. This is consistent with the litterature (14, 15, 17) and suggests that automatic gaze shifting is equally developed in PC and TC. Notably, neither PC nor TC responded faster when a cue oriented their attention to the location of the upcoming target, suggesting that orienting may not be yet fully developed in PC or TC aged 6–7 years (26). In addition, no influence of the body mobility condition was found on the function of orienting, neither in PC nor in TC. A decrease in body mobility may be observed when participants have to precisely displace and orient their gaze (66). If a decrease in body mobility and gaze stabilization is beneficial for the function of orienting, it can explain why a more challenging posture than the standing posture and a free-to-move condition did not help children to orient their attention better.

### Age

The small sample size and the limited age range of our study might have prevented the observation of a significant improvement in attentional function with age. In fact, Mezzacappa (26) showed that age was associated with improved use of alerting cues and orienting cues and improved dealing with interference, among 249 children between 5 and 7 years. Notably, we observed an overall faster reaction time for children older than 80 months compared with those who were younger, consistent with improvement of processing with age. A clinical implication is that infants born at the end of a calendar year might show slightly lower performances than their counterparts born at the beginning of the same calendar year.

### Strengths and limitations

The use of the ANT-c enabled testing of the attentional functions both together and separately. We showed different influences on body mobility and prematurity depending on the attention function studied.

This study has limitations. First, the relatively small sample size and the exclusion criteria may limit the generalization of the results to the entire PC population. However, these results may apply to PC attending normal schools in their appropriate grade level. Second, mothers of PCs had a generally lower social level, but this reflects the higher rate of PC within a lower socioeconomic background (67). Third, we did not evaluate the IQ of the children at the time of the study. This was mainly due to the need for limitation of the experiment duration. An excessive children’s tiredness would have been an important bias for evaluating attentional performance. However, it was confirmed during the interview with parents and the clinical exam by the trained pediatrician that none of the children had cognitive, developmental, or motor disorders. Finally, regarding the free-to-move condition, approximately 80% of the children moved their body parts for less than 5 s per minute (Supplementary Figure 2 in Supplementary material). As stated previously, one may speculate that the level of motor activity in our children was not sufficient to improve alertness or executive performance. One explanation may be that all children needed to stabilize the position of their head and their gaze in order to correctly perform the requested visual task, reducing their motor activities. Furthermore, despite the interest in using a head-mounted display in order to keep the eyes and the target constant, it is possible that this device reduced the spontaneous motricity of the children due to their infrequent use in ecological situations and the lack of visual information about the environment. Evaluating the influence of body mobility on other cognitive tasks, using other devices and modalities (e.g., auditive tasks), may be interesting to confirm this assumption. In addition, it would be interesting in future studies to use devices, such as accelerometers or force plates, to more precisely evaluate the levels of motor activity and postural control among PC and TC.

## Conclusion

This study supports the importance of including body activity analysis when evaluating cognitive performance. It showed that it is important (1) to study more precisely and separately the three attentional functions that may have different development timings in children born at term or prematurely and (2) to include observation of their motor activities in the evaluation.

Our results showed that executive control decreased in the standing-still posture compared with the sitting-still posture among PC, contrasting with TC and adult behavior. The clinical implication may be that PC would alter their cognitive performance and learning ability when asked to stand up at school contrary to term-born children. Taking into account body posture and mobility during classes might prevent those vulnerable children from altering their cognitive function. It would improve their learning ability and academic achievement. To confirm this assumption, it would be interesting to perform a more precise analysis of PC postural activity while performing cognitive tasks.

## Data availability statement

The raw data supporting the conclusions of this article will be made available by the authors, without undue reservation.

## Ethics statement

The studies involving human participants were reviewed and approved by Comité de Protection des Personnes Sud-Est III Ethics Committee (2017–010 B). Written informed consent to participate in this study was provided by the participants’ legal guardian/next of kin.

## Author contributions

JR, J-MH, HC, SC, and HD contributed to the conception and design of this study. IH performed a clinical examination for each child. HD followed the yearly evaluation of the PC. JR, AMJH, and HC conducted the experiments and collected the data. JR and HC analyzed children’s movements. JR, SC, HC, and J-MH processed the data and performed the statistical analysis. JR, HC, SC, IH, and J-MH discussed the results and wrote the manuscript. All authors contributed to this study and approved the submitted version.
