# Supplementary material for: Influence of body mobility on attention networks in school-aged prematurely born children: A controlled trial
Source: Front Pediatr. 2022 Sep 8;10:928541. doi: 10.3389/fped.2022.928541 (PMC9492848; doi:10.3389/fped.2022.928541)
Supplement: Supplementary file 1 [file Data_Sheet_1.PDF]

Figure 1s (supplemental material). Attention Effect for PC and TC in the Three Body Mobility Conditions

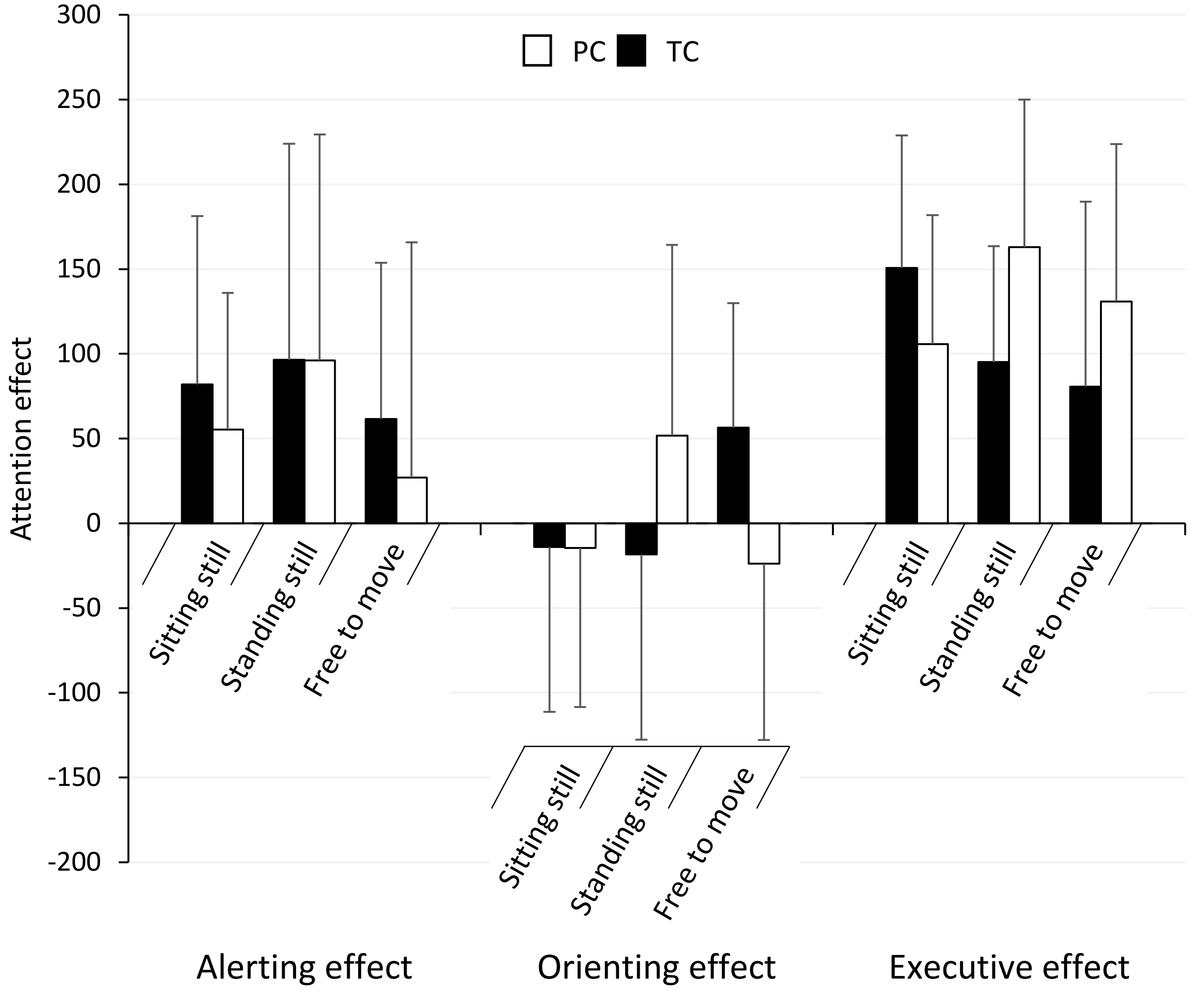

Abbreviations: TC, term-born children; PC, prematurely born children.
