# Supplementary material for: Influence of body mobility on attention networks in school-aged prematurely born children: A controlled trial
Source: Front Pediatr. 2022 Sep 8;10:928541. doi: 10.3389/fped.2022.928541 (PMC9492848; doi:10.3389/fped.2022.928541)
Supplement: Supplementary file 2 [file Data_Sheet_2.PDF]

Figure 2s (supplemental material). Rate of TC and PC Movement in the Three Body Mobility Conditions

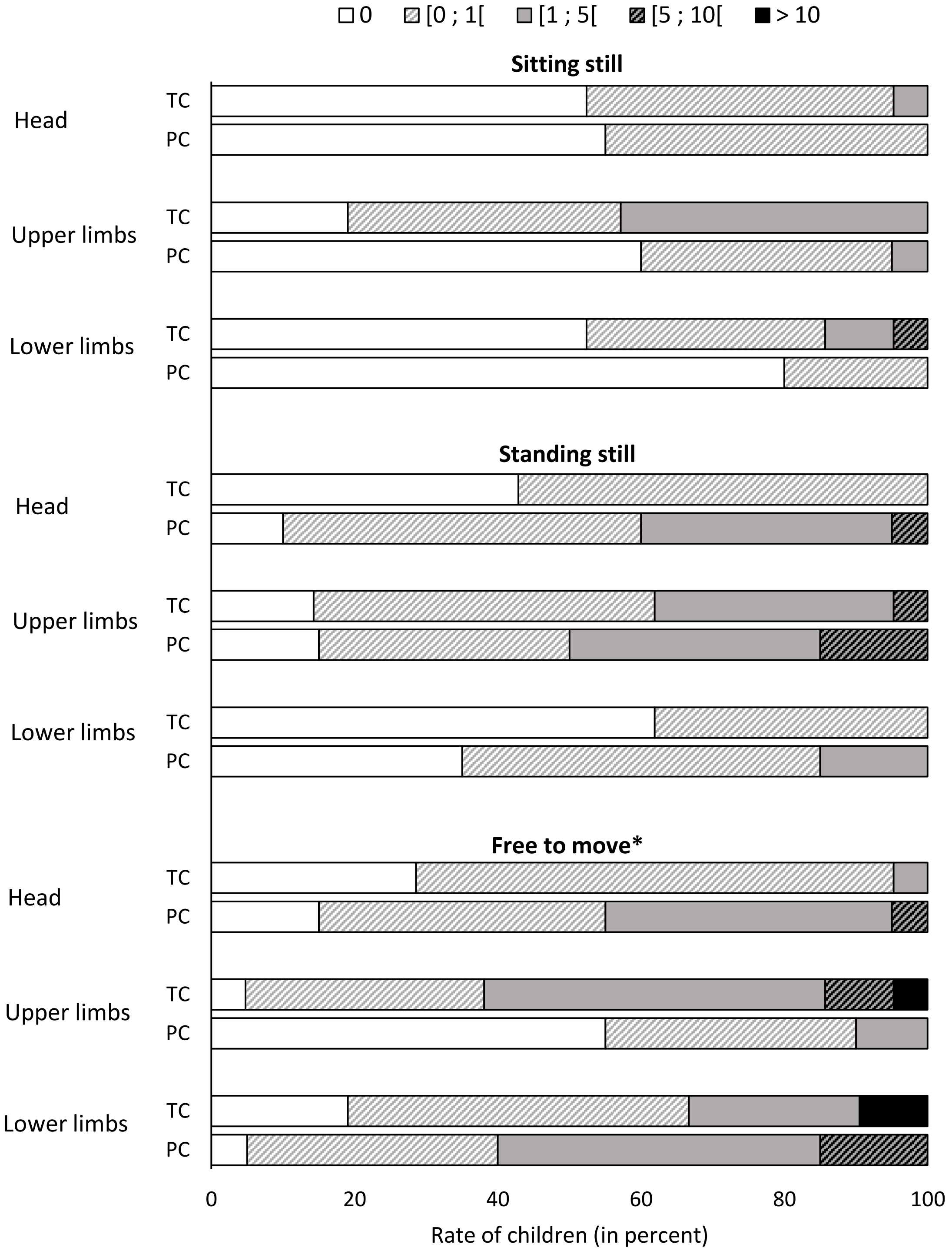

Rates shown include not moving (0), and moving for 0-1, 1-5, 5-10, and >10 seconds per minute, for different body parts, under the three body mobility conditions.

***** 21 TC and 18 PC chose to sit down at least one time, and 11 TC and 4 PC exclusively sat; 3 TC and 2 PC kneeled and 4 TC and 4 PC sat cross-legged. Six TC and 8 PC adopted a standing posture at least one time, and 6 TC and 8 PC lay down at least one time (2 TC and 2 PC lay down on the stomach and the others on their back).

Abbreviations: TC, term-born children; PC, prematurely born children.
